# Croup

**Published:** 1880-10

**Authors:** 


**CROUP** is an inflammation of the inner surface of the wind-pipe. Inflammation implies heat, and that heat must be subdued or the patient will inevitably die. If prompt efforts are made to cool the parts in case of an attack of croup, relief will be as prompt as it is surprising and delightful. All know that cold applied to a hot skin cools it, but all do not as well know and understand, that hot water applied to an inflamed skin will as certainly cool it off. Hence the application of ice-cold water with linen cloths, or of almost boiling water with woolen flannel, are very efficient in the cure of croup. Take two or three pieces of woolen flannel of two folds, large enough to cover the whole throat and upper part of the chest, put these in a pan of water as hot as the hand can bear, and keep it thus hot, by adding water from a boiling tea-kettle at hand; let two of the flannels be in the hot water all the time, and one on the throat all the time, with a dry flannel covering the wet one, so as to keep the steam in to some extent; the flannels should not be so wet, when put on, as to dribble the water, for it is important to keep the clothing as dry as possible, and the body and feet of the child comfortable and warm. As soon as one flannel gets a little cool, put on another hot one, with as little interval of exposure as possible, and keep up this process until the doctor comes, or until the phlegm is loose, the child easier, and begins to fall to sleep; then gently wrap a dry flannel over the wet one which is on, so as to cover it up thoroughly, and the child is saved. When it wakes up, both flannels will be dry. The same results will follow if cold water is used, the colder the better; the cloths should be of muslin or linen and of several folds thickness, large enough to cover the whole throat and the upper part of the breast.

